# Transcriptomic Identification of Core Regulatory Genes for Higher Alcohol Production in *Saccharomyces cerevisiae* at Different Sugar Concentrations in Wine Fermentation

**DOI:** 10.3390/foods14091476

**Published:** 2025-04-23

**Authors:** Lu Chen, Xiaona Ren, Yanan Wang, Dongshu Hao, Yanying Liang, Yi Qin

**Affiliations:** 1 College of Enology, Northwest A&F University, Yangling 712100, China; 2 Xinjiang Zhang Yu Ba Bao Baron Winery Co., Ltd., Shihezi 832000, China; 3 National Forestry and Grassland Administration Engineering Research Center for Viti-Viniculture, Yangling 712100, China

**Keywords:** higher alcohols, sugar concentration, transcriptomics, *saccharomyces cerevisiae*

## Abstract

Higher alcohols are significant flavor compounds in wine, and the elevated sugar content in grape raw materials has a substantial impact on wine quality. This study investigates the effect of high sugar content on the production of higher alcohols in wine and elucidates the underlying mechanisms through transcriptome analysis. The results indicate that sugar concentrations of 240 g/L and 280 g/L lead to increases in higher alcohol content of 17% and 24%, respectively. Transcriptome sequencing was employed to analyze differentially expressed genes at various fermentation stages, which resulted in the identification of the *GRE3* gene. It was determined that the expression level of *GRE3* significantly influences higher alcohol content. Knocking out *GRE3* using molecular methods led to a notable 17.76% decrease in higher alcohol yield at a sugar concentration of 240 g/L, representing a novel finding not previously documented in the literature. This research provides valuable insights into the influence of high-sugar grape materials on the production of higher alcohols by *Saccharomyces cerevisiae* and the associated mechanisms.

## 1. Introduction

Higher alcohols are the byproducts of *Saccharomyces cerevisiae* fermentation [1]. Alcohols are important derivatives of carbon and amino acid metabolism in yeast [1,2,3]. As important volatile compounds, they influence wine quality [4,5]. An appropriate quantity of higher alcohols can enhance the wine body, create a mellow taste, and contribute positively to the complexity of the wine aroma [6]. However, an excessively high alcohol content can disrupt the flavor balance, leading to an unpleasant experience characterized by spiciness and bitterness [7,8]. Moreover, it may cause headaches, nausea, and other intoxication symptoms [9]. Consequently, researchers have focused on controlling the formation of higher alcohols to achieve the desired amounts during the winemaking process.

The production of higher alcohols in wine is related to various fermentation conditions such as the type of yeast [10], content and type of assimilable nitrogen [11], sugar content, fermentation temperature [12], initial pH of the fermentation substrate, and dissolved oxygen content [13]. Among these factors, the sugar content of the fermentation medium is particularly significant. It is generally believed that 75% of the higher alcohol synthesis is derived from the Harris pathway, and 25% is formed by amino acid decarboxylation reduction (Ehrlich pathway) [14]. In 1953, Harris found that higher alcohols are primarily derived from keto acid metabolism during glucose metabolism [15]. In this pathway, α-keto acid, a product of glucose metabolism, is further decarboxylated and dehydrogenated to produce higher alcohols under the catalysis of the corresponding enzymes. Therefore, the sugar content of grapes can directly affect the levels of higher alcohols in wine.

In recent years, global warming has led to the excessive accumulation of sugar in grape berries [16]. Excess sugar promotes the Harris pathway in *S. cerevisiae* and increases the content of higher alcohols in wine [17]. To effectively control the synthesis of higher alcohols by yeast via the Harris pathway, it is necessary to comprehensively understand the Harris pathway of higher alcohols and its regulatory mechanisms. These mechanisms have yet to be fully elucidated.

Transcriptomic technologies have demonstrated revolutionary potential in the field of winemaking. By analyzing the dynamic gene expression of *S. cerevisiae* during fermentation, researchers can accurately identify key gene clusters associated with the synthesis of flavor compounds and responses to environmental stress [18,19]. Ma et al. [20] employed RNA-seq technology to reveal that the downregulation of ARO9 and ARO10 genes during the alcoholic fermentation stage reduces the synthesis of higher alcohols, while the upregulation of ALDH and acetyl-CoA promotes the accumulation of esters. Drozdova et al. [21] conducted a transcriptome analysis that revealed a relationship between differential gene expression at varying lactic acid concentrations and the inhibition of *S. cerevisiae* growth, thereby illuminating the influence of pH on this process. Such studies not only enhance our understanding of the metabolic regulatory network of *S. cerevisiae* but also provide molecular targets for process optimization.

In this study, transcriptomic analysis was conducted to explore the effect of sugar concentration on the production of higher alcohols and to identify core regulatory genes for higher alcohol production by *S. cerevisiae* at different sugar concentrations during different phases of wine fermentation. This study enhances our understanding of the metabolic pathways of higher alcohols and offers significant insights into the cultivation of *S. cerevisiae* that produces lower levels of these compounds. This is particularly relevant for fermentation systems that operate under varying sugar concentrations in the brewing industry.

## 2. Materials and Methods

### 2.1. Yeast Strains

The yeast used in this study was *S. cerevisiae* LFE1225, which produces a high yield of higher alcohols. Details of the strains and plasmids used are listed in Supplementary Table S1.

Yeast extract–peptone–dextrose (YPD) solid medium contained 20 g/L glucose, 20 g/L peptone, 10 g/L yeast extract, and 20 g/L agar powder.

Luria–Bertani (LB) medium contained 10 g/L NaCl, 10 g/L peptone, 5 g/L yeast extract, and 20 g/L agar powder.

Synthetic dextrose without uracil (SD-URA) medium contained 26.7 g/L minimal SD base, 1.29 g/L dropout supplement-URA, and 20 g/L agar powder.

Media used for seed culture and fermentation was prepared in accordance with the Triple M chemically defined media (CDM) [22] preparation method and sterilized by 0.22 µm filtration. The initial pH value is 3.2.

### 2.2. Fermentation Conditions

Seed cultures of yeast cells were cultured at 28 °C and 150 rpm for 24 h. The inoculum density was approximately 5 × 10^5^ colony-forming units/mL. Based on the CDM containing 200 g/L and glucose to fructose ratios of 1:1, 40 g/L, and 80 g/L were added to prepare CDM under three sugar conditions (200 g/L, 240 g/L, and 280 g/L).

### 2.3. Determination of the Basic Physicochemical Parameters

High-temperature sterilization is conducted using 500-milliliter conical flasks. The CDM is prepared at a volume of 300 milliliters per flask. After sterile filtration, the seed culture is introduced, and the neck of the bottle is sealed with sealing film for fermentation. Fermentation was carried out in a stationary incubator maintained at 25 °C. Every 24 h, the fermentation flask was weighed until the CO_2_ weight loss was less than 1 g, which was considered as the end of fermentation. The conical flask was weighed every 24 h to detect weight loss of CO_2_. A fermentation curve was drawn with time as the abscissa and CO_2_ weight loss rate as the ordinate. The residual sugar in the medium was detected using the 3,5-Ddinitrosalicylic acid method until the residual sugar was less than 4 g/L, which was regarded as the end of fermentation.

High-performance liquid chromatography (HPLC) was used to determine the concentrations of residual sugars, glycerol, ethanol, and organic acids at the end of the fermentation. An HPX-87H hydrogen ion column (Bio-rad, Shanghai, China) (300 mm × 7.8 mm) equipped with a DAD UV and RID-type differential detector was used as the chromatographic column. The mobile phase was a 5 mM H_2_SO_4_ (HPLC-grade) aqueous solution set at a flow rate of 0.6 mL/min. The column temperature was maintained at 60 °C, and the detection wavelengths were set at 210 nm and 254 nm. The final injection volume was 20 μL. Organic acid standards were prepared as 100 g/L tartaric acid, 100 g/L lactic acid, 500 g/L malic acid, 500 g/L citric acid, and 500 g/L pyruvic acid standard masterbatches. Different amounts of the reserve solution were pipetted into 1 mL of the system to prepare mixed master batches. The mixed master batch was subjected to gradient dilution, followed by injection into a liquid chromatograph. Relevant organic acids were identified based on their respective peak retention times after 30 min of operation. Standard curves were created using various concentrations of the standards as horizontal coordinates and the corresponding peak areas as vertical coordinates. Linear regression equations were derived from these curves. Sample Preparation: Begin by adding 1 mL of simulated wine sample to a 5 mL centrifuge tube, followed by the addition of 3 mL of commercially available purified water (Hangzhou Wahaha Group Co., Ltd., Hangzhou, China). Dilute the mixture to achieve a final sugar concentration of less than 2 g/L. Thoroughly mix the solution by vortexing for 30 s, filter it through a 0.22 μm filter membrane, and finally seal it in a 1.5 mL vial. The total quantities of glycerol were measured using an Enology Y15 (BioSystems, Barcelona, Spain). Higher alcohols were detected using gas chromatography-mass spectrometry [23].

### 2.4. Transcriptome Sequencing

Based on the fermentation curves of strain LFE1225 at the three sugar concentrations, we defined five phases: initial (0 h, FI), early- (24 h, early), mid- (72 h, middle), late- (360 h, late), and end of fermentation (end). Samples were collected at three time points (early, middle, and late fermentation) for transcriptome sequencing. Yeast cells were sampled from the CDM (50 mL) and centrifuged at 9000 rpm and 4 °C for 30 s. The supernatant was discarded, and the cells were mixed with 1.5 mL of phosphate-buffered saline. The mixture was shaken and centrifuged at 9000 rpm and 4 °C for 30 s. After discarding the supernatant, the cells were quickly frozen in liquid nitrogen and stored at −80 °C until RNA extraction.

Total RNA was extracted using a TRIzol reagent kit (Invitrogen, Carlsbad, CA, USA) according to the manufacturer’s protocol. RNA quality was assessed using an Agilent 2100 Bioanalyzer (Agilent Technologies, Palo Alto, CA, USA) and verified using RNase-free agarose gel electrophoresis. After the total RNA was extracted, eukaryotic mRNA was enriched using oligo (dT) beads, whereas prokaryotic mRNA was enriched by removing rRNA using the Ribo-ZeroTM Magnetic Kit (Epicentre, Madison, WI, USA). The enriched mRNA was then fragmented into short fragments using a fragmentation buffer and reverse-transcribed into cDNA using random primers. Second-strand cDNA was synthesized using DNA polymerase I, RNase H, dNTP, and buffer. The cDNA fragments were purified using the QiaQuick polymerase chain reaction (PCR) extraction kit (Qiagen, Venlo, The Netherlands), end-repaired, an A base added, and ligated to Illumina sequencing adapters. The ligation products were size-selected by agarose gel electrophoresis, PCR-amplified, and sequenced using an Illumina NovaSeq 6000 (Gene Denovo Biotechnology Co., Guangzhou, China). The raw sequence data generated in this study have been deposited in the Genome Sequence Archive database of the BIG Data Center (http://gsa.big.ac.cn/index.jsp, accessed on 31 July 2023) under the GSA number CRA011957.

### 2.5. Transcriptome Analysis

Raw reads were processed using FASTP to obtain clean reads, which were analyzed using HisAT2 software (version 2.0.3.12) for comparison with the reference genome of *S. cerevisiae* S288C (https://www.ncbi.nlm.nih.gov/datasets/genome/GCF_000146045.2/, accessed on 15 June 2023). Fragments per kilobase of transcript per million mapped reads were used as the unit of gene expression quantification. Gene abundance was quantified using RSEM 1.3.3 software. Standardized read counts were performed using edge R 4.3.1 and Deseq2 1.38.0 software to screen for significant differentially expressed genes (DEGs) (false discovery rate [FDR] < 0.05, |log2 fold change [FC]| > 1). DEGs were mapped to terms in the Kyoto Encyclopedia of Genes and Genomes (KEGG) and Gene Ontology (GO) databases, with FDR ≤ 0.05. Based on the results of differential analysis, the target genes were chosen to draw a heatmap.

### 2.6. Plasmid Construction and Recombinant Strain Screening

The TAATAA terminator was inserted into the target gene (*GRE3*) in the parent strain LFE1225 using CRISPR/Cas9 technology to eliminate its function. The PCR primers used are listed in Supplementary Table S1. Primers were based on the *S. cerevisiae* S288c genome (http://www.ncbi.nlm.nih.gov/). The PRCC-K plasmid was used as a template, and sgF1 and sgF2 were used as primers to amplify and guide RNA expression box P1. Using the genome of the parent strain as a template, primers G-F, G-Rm, G-Fm, and G-R were used to amplify the upstream and downstream homologous fragments P2 and P3, respectively. Fragments P1, P2, and P3 were ligated into the PRCC-K plasmid using a one-step cloning kit and transferred into *Escherichia coli*. Recombinant plasmid screening was performed using an LB medium containing 1 mg/mL ampicillin, and colonies containing the correct recombinant plasmid were confirmed using PCR. Homologous recombination of the homologous fragments and the yeast genome was achieved by transferring the recombinant plasmids into yeast using the lithium acetate/single-stranded DNA/polyethylene glycol method [24]. A solid YPD medium containing 2 mg/mL G418 was used for the initial screening of transformants, and accurate integration of terminators into individual colonies was confirmed by PCR and sequencing. Plasmids were discarded by sub-culturing. Ultimately, the correct single colony was the constructed recombinant knockout strain.

*GRE3* was amplified using primers G-A and G-D using the genome of *S. cerevisiae* LFE1225 as a template. The *GRE3* fragment was linked to the pY26 plasmid using a one-step cloning kit to construct a gene overexpression vector. Plasmid selection was performed using LB/ampicillin medium and PCR. The plasmids were transferred into yeast to overexpress *GRE3*.

### 2.7. Statistical Analysis

Data were processed using SPSS 20.0 and expressed as mean values ± standard deviation. The same software was used to perform a one-way analysis of variance (ANOVA) to determine significant differences between the experimental and control groups. The confidence interval for one-way ANOVA was set at 95%. Venn diagrams, heat maps, GO, and KEGG analyses were performed using OmicShare, a free online platform for data analysis (https://www.omicshare.com/tools, accessed on 29 June 2023). The remaining figures were plotted using GraphPad Prism 9 software (GraphPad Software, San Diego, CA, USA).

## 3. Results

### 3.1. Fermentation Performance at Three Sugar Concentrations

At the three sugar concentrations, *S. cerevisiae* LFE1225 began fermenting within 24 h and reached the maximum fermentation rate 72 h after inoculation (Supplementary Figure S1A). A high sugar concentration corresponded to a high fermentation rate, and at a concentration of 280 g/L, the fermentation time was extended by 5–6 days compared to a low sugar concentration.

The trends of ethanol, glycerol, and acetate exhibit similar patterns across varying sugar concentrations, all showing a positive correlation with sugar concentration (Table 1 and Supplementary Figure S1C). Upon fermentation completion, ethanol concentrations under high-sugar conditions (240 g/L and 280 g/L) increased by 24% and 34%, glycerol content by 23% and 43%, and acetate content by 44% and 72%, respectively, compared to low sugar concentrations (200 g/L). Total acidity initially increases during the fermentation stage, followed by a subsequent decrease. In contrast, the levels of citric acid, succinic acid, and lactic acid remain unaffected by changes in sugar concentration. The pyruvate and malate content initially increased and then decreased. As the sugar content increased, pyruvate content continued to decrease, whereas malate content showed no significant differences among the three sugar concentrations at the end of fermentation.

### 3.2. Effect of Sugar Concentration on the Higher Alcohol Production by S. cerevisiae

**Figure 1:**
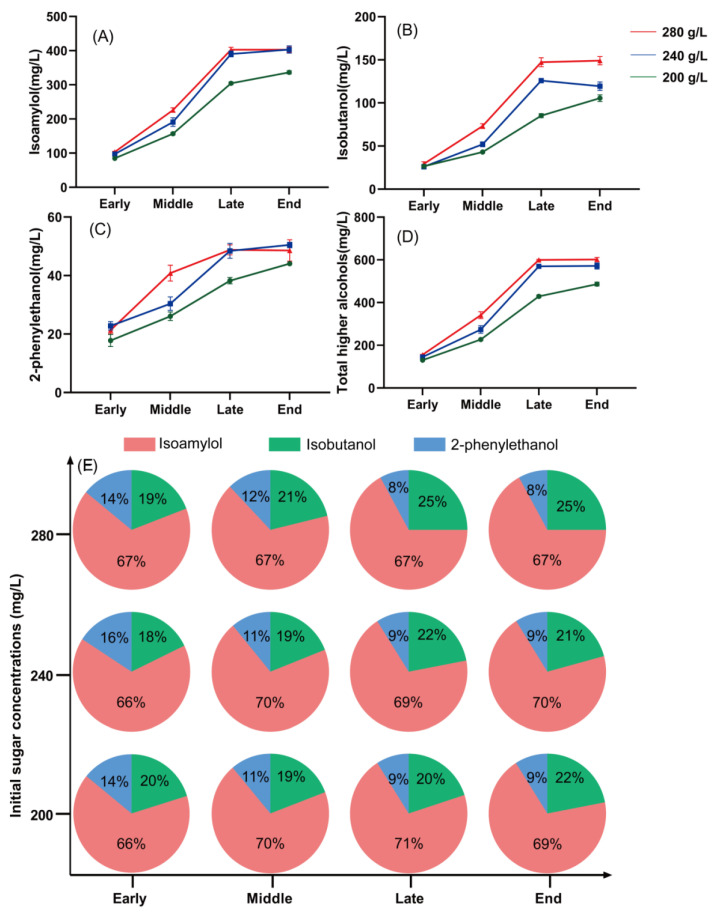
The concentrations of isobutanol, isoamylol, 2-phenylethanol, and total higher alcohols at different sugar concentrations throughout the fermentation process by LEF1225. Early fermentation (24 h, FA). Mid-fermentation (72 h, FB). Late fermentation (360 h, FC). The end of fermentation (FD). (**A**): Isoamylol. (**B**): Isobutanol. (**C**): 2-phenylethanol. (**D**): Total higher alcohols. (**E**): Proportion of different higher alcohols in different initial sugar concentrations.

At all fermentation periods, the contents and percentages of isoamylol, isobutanol, and 2-phenylethanol produced were measured at the different sugar concentrations (Figure 1). Sugar concentration affected the yield of higher alcohols from LFE1225. All higher alcohol contents increased with increasing sugar concentrations. Compared with the 200 g/L low-sugar fermentation system, the total higher alcohol contents of the high-sugar (240 and 280 g/L) fermentation systems were significantly different, increasing by 11% and 20% in the early (FA), 21% and 50% in the middle (FB), 33% and 40% in the late (FC), and 17% and 24% in the end (FD) growth stages, respectively. Notably, at sugar concentrations of 240 g/L and 280 g/L, the concentration of higher alcohols displayed no significant changes from the late fermentation stage until completion. Isoamylol and isobutanol contents were significantly affected by sugar concentration. Isobutanol was significantly affected by sugar concentration during all four periods, whereas isoamylol alcohol was more significantly affected by sugar concentration only during the middle fermentation period. The ratio of 2-phenylethanol was not significantly affected by sugar concentration.

### 3.3. Transcriptional Response to Sugar Concentration

Samples from the FA, FB, and FC growth stages were collected at sugar concentrations of 200, 240, and 280 g/L, respectively. We performed RNA-seq analysis of the *S. cerevisiae* LFE1225 transcripts under different high-sugar fermentation conditions. The percentage of sequences mapped to the genome under the nine conditions ranged from 90.40% to 96.52%. These data suggested that the throughput and sequencing quality were sufficiently high for further analysis (Supplementary Table S2).

**Figure 2:**
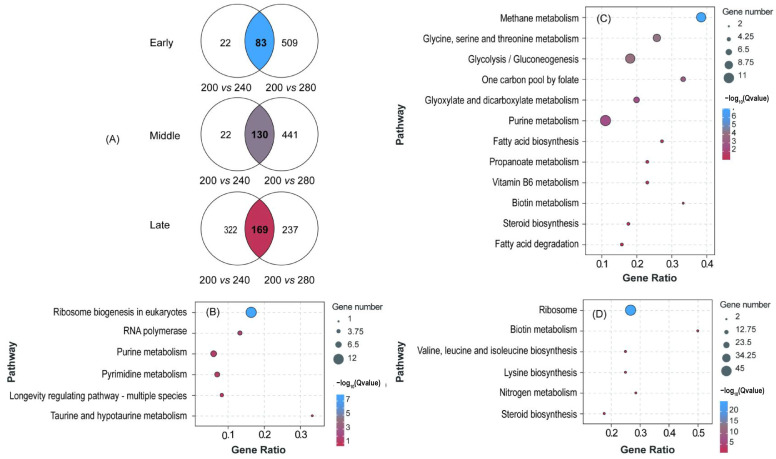
DEGs at different sugar concentrations and KEGG enrichment. (**A**) The Venn diagrams show the number of significantly different genes in the comparison groups of sugar concentration changes during early, middle, and late fermentation periods. (**B**–**D**) represent KEGG pathway enrichment maps of early, middle, and late differential genes in (**A**).

Using a Venn diagram analysis (Figure 2A), in the early phase, 83 genes responded to both sugar concentration treatments. These genes were mainly involved in ribosomal biogenesis in eukaryotes and in purine and pyrimidine metabolism (Figure 2B). In the middle phase, 130 genes responded to both sugar treatments. Functional enrichment was mainly concentrated in glycine, serine, threonine, and purine metabolism; glycolysis; purine metabolism; and fermentation-limiting factors of nutrients such as one carbon pool by folate, Vitamin B6 metabolism, biotin metabolism, and steroid biosynthesis (Figure 2C). In the late phase, 169 genes responded to both sugar treatments. These were mainly concentrated in ribosome and amino acid metabolism, such as valine, leucine, isoleucine, and lysine biosynthesis (Figure 2D).

### 3.4. Genes Related to Higher Alcohol Metabolism

**Figure 3:**
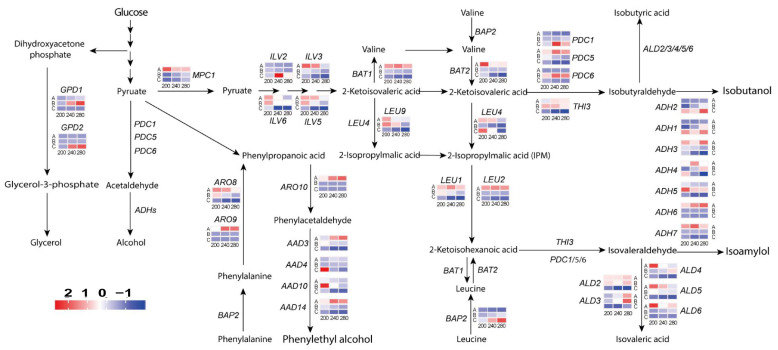
Expression of genes related to higher alcohol synthesis pathways in yeast cells at different sugar concentrations and fermentation durations. The gene heat map from left to right represents the gene expression under the fermentation conditions of sugar concentrations of 200 g/L, 240 g/L and 280 g/L, respectively, and the gene expression in the early, middle, and late stages of fermentation from top to bottom. Letters A, B, and C represent the early, mid, and late phases of fermentation, respectively.

The 37 target genes were analyzed in combination with the isoamylol, isobutanol, and 2-phenylethanol pathways, which are involved in higher alcohol metabolism (Figure 3). Under all sugar conditions, *MPC1*, *BAT1/2*, *ARO8/9/10*, *LEU1/2*, *ILV3/5/6,* and *ALD4/5/6* exhibited relatively high gene-expression levels during the pre-fermentation phase. However, gene expression decreased during the late fermentation phase. During the early fermentation stage, the gene expression levels of *AAD3/14*, *ARO9*, *ARO10*, and *ADH6* were higher than those observed at 200 g/L sugar, particularly under the high-sugar condition of 280 g/L. In contrast, *LEU4/9*, *ARO8*, *ILV3/5/6*, *BAT2*, and *ALD4/5/6* showed significantly lower gene expression levels. In the middle stage of fermentation, which is also the stage of fastest synthesis of higher alcohols, the expression levels of *GPD1*, *ALD3*, and *PDC6* were relatively high, especially at 280 g/L sugar. At the end of fermentation, both *ADH1* and *ADH2* were highly expressed under the various sugar conditions. *PDC1* and *BAP2* displayed elevated expression levels only under high sugar concentrations of 240 and 280 g/L, whereas *LEU4* and *AAD4* demonstrated increased expression levels only under 200 g/L sugar conditions.

### 3.5. Venn Diagram Identification of Key Gene

The genes contained at the intersection of the FA, FB, and FC periods showed a significant response to changes in sugar concentration. By merging the intersections of the three periods to form a new gene set, the target DEGs responded to both the fermentation period and sugar concentration changes.

**Figure 4:**
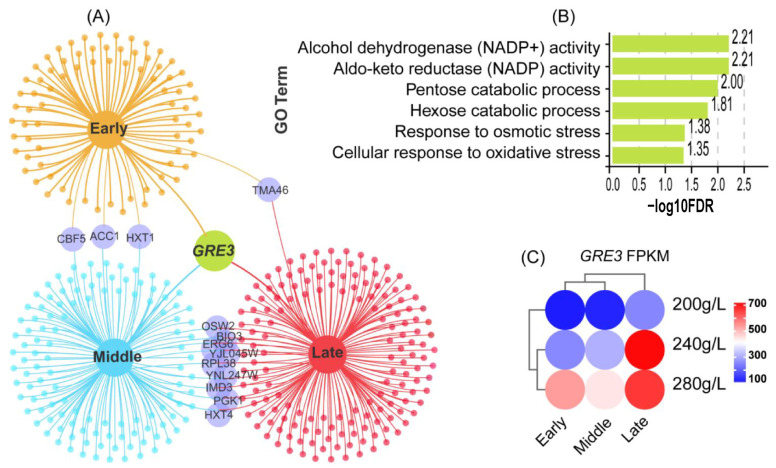
Expression of *GRE3* gene in different sugar concentrations and fermentation periods. (**A**) Mining of the *GRE3* gene. Cross-analyzing DEGs enriched for different sugar concentrations in the three periods, we screened out the common differential gene GER3 in three periods. (**B**) GO enrichment analysis. (**C**) Expression of *GRE3* genes in different sugar concentrations and fermentation periods.

There was one DEG, *GRE3*, whose expression levels were significantly affected by changes in sugar concentrations during the FA, FB, and FC periods (Figure 4). *GRE3* encodes an NADPH-dependent aldose reductase with broad substrate specificity. The GO terms found that *GRE3* was associated with several terms, such as alcohol dehydrogenase (NADP^+^) activity, aldo-keto reductase (NADP) activity, and the pentose catabolic process. Further validation is required to determine the regulatory effects on higher alcohols.

### 3.6. Production of Higher Alcohols in GRE3 Recombinant Strains

**Figure 5:**
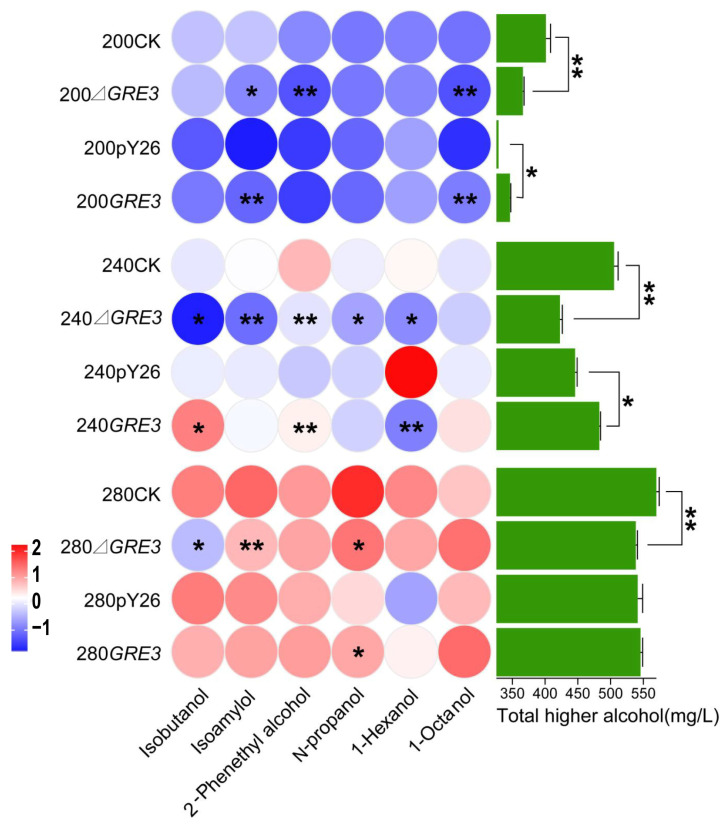
Heat map of higher alcohol data of *GRE3* gene recombinant strains. The original strain LFE1225 was used as the control strain for gene deletion, and the strain transformed into empty vector pY26 was used as the control strain for gene overexpression. The darker the color, the higher the content of this substance in that condition. The far right shows the total higher alcohol production of each strain (**: 0.001 < *p* ≤ 0.01; *: 0.01 < *p* ≤ 0.05).

The recombinant strains in fermentation media with 200, 240, and 280 mg/L sugar concentrations were studied, and the effects of *GRE3* genes on higher alcohol production were analyzed by comparison with the parental strain. The yields of higher alcohols after fermentation are shown in Figure 5. Compared with the parental strain, the total higher alcohols of strain Δ*GER3* (deletion of *GRE3* gene) were significantly decreased by 9.59% (38.69 mg/L), 17.76% (90.19 mg/L), and 6.08% (34.72 mg/L) in 200 mg/L, 240 mg/L, and 280 mg/L sugar concentrations, respectively. The isoamylol and 2-phenethyl alcohol contents decreased significantly at a sugar concentration of 200 g/L, and those of isobutanol, isoamylol, 2-phenethyl alcohol, n-propanol, and 1-hexanol decreased significantly at 240 g/L. At 280 g/L, the isobutanol, isoamylol, and n-propanol contents decreased significantly.

Compared with the control strain (carrying the pY26 plasmid), the production of higher alcohols in the *GRE3* overexpression strain increased by 5.02% (16.64 mg/L) and 8.50% (37.67 mg/L) at sugar concentrations of 200 and 240 g/L, respectively, whereas the contents of total higher alcohols did not change significantly at 280 g/L. The isoamylol and 1-octanol contents increased significantly at 200 g/L, isobutanol and 2-phenethyl alcohol increased significantly at 240 g/L, and the hexanol content decreased significantly. At 280 g/L, only the n-propanol content increased significantly.

## 4. Discussion

During wine fermentation, the initial carbon source concentration is critical for yeast growth kinetics [25,26] and has a significant impact on the metabolites that affect wine quality [27,28]. This study utilized RNA-seq high-throughput sequencing technology to examine the transcriptional characteristics of the LEF1225 strain across three distinct sugar concentrations and three fermentation stages. The analysis revealed significant changes in the expression levels of genes associated with various higher alcohol metabolic pathways. Differentially expressed genes at different fermentation stages were analyzed collectively through transcriptome sequencing, leading to the identification of the *GRE3* gene. Functional validation indicated that variations in the expression level of *GRE3* significantly influence higher alcohol content.

### 4.1. Effects of High Sugar on Higher Alcohols and Other Metabolites of S. cerevisiae

Sugar concentration in the fermentation medium significantly impacts the formation of higher alcohols. The ability of *S. cerevisiae* to produce these alcohols is influenced not only by the type of carbon source but also by its concentration, which directly affects alcohol yield. Studies indicate that the content of higher alcohols increases as the initial fermentation carbon-to-nitrogen (C/N) ratio declines [29]. In this study, at sugar concentrations of 240 g/L and 280 g/L, the concentration of higher alcohols remained relatively stable from the late fermentation stage to completion. This stability is attributed to the rising concentration of the carbon source, which subsequently elevates the C/N ratio and inhibits the synthesis of higher alcohols.

Sugar is the main energy source for yeast metabolism and is the main source of yeast metabolite production. High-sugar conditions (280 g/L) did not inhibit yeast fermentation performance (Supplementary Figure S1A) but instead increased the activity. In previous studies, the most severe inhibition of yeast growth was observed at a sugar concentration of 100 g/L fructose [30], which was likely related to the yeast strains [31,32]. Glycerol is a representative stress metabolite produced during the fermentation process of hyperosmotic-tolerant yeast. The hydroxyl groups in glycerol confer significant hydrophilicity, enabling it to effectively regulate cellular osmotic pressure and protect cells from damage associated with osmotic fluctuations [33]. Research indicates that under high-sugar conditions, the secretion of glycerol by *S. cerevisiae* increases, allowing it to maintain osmotic pressure in environments with sugar concentrations not exceeding 700 g/L. This elevation in glycerol production may be attributed to enhanced gene expression related to glycerol synthesis in response to high sugar stress within this concentration range [34]. In this study, glycerol content exhibited a significant increase with rising sugar concentrations (Supplementary Figure S1), while the expression of genes associated with glycerol synthesis, *GPDs*, also markedly increased (Figure 3). These results are consistent with findings reported in previous research [35].

The primary organic acids in wine include tartaric acid, malic acid, lactic acid, acetic acid, succinic acid, citric acid, and pyruvic acid. During fermentation, the concentration of malic acid initially increases and then decreases. This trend occurs because, in the early stages of fermentation, the metabolism of organic acids and sugars generates a higher quantity of malic acid, resulting in an initial rise in its concentration, which subsequently declines due to metabolic processes or utilization. Similarly, the concentration of citric acid exhibits a pattern of initial increase followed by a decrease, with differences becoming negligible by the end of fermentation. This pattern indicates that citric acid, malic acid, and succinic acid undergo mutual transformation as intermediates in the tricarboxylic acid cycle. Pyruvic acid, another intermediate in alcoholic fermentation, forms at the onset of the process and decreases by its conclusion [36]. Total acidity shows a trend of initially increasing followed by a subsequent decrease. High sugar concentrations can induce *S. cerevisiae* to produce elevated levels of total acids and volatile acids, likely due to the yeast’s adaptation to the osmotic pressure created by the high sugar environment. This adaptation results in enzymatic reactions occurring within the cells [37], leading to an increase in total acidity during the early stages of fermentation. Malic acid constitutes over 90% of the total acidity [38], and its marked decline after the early stages of fermentation subsequently contributes to the overall decrease in total acidity.

### 4.2. Transcriptome Explanation of the Effect of High Sugar on Production of Higher Alcohols by S. cerevisiae

In the initial stage of fermentation, the concentration of amino acids in the fermentation liquid is relatively high, which can inhibit the yeast cells’ Ehrlich pathway through feedback mechanisms. During this period, yeast preferentially degrades exogenous amino acids into corresponding higher alcohols via the Ehrlich pathway, thereby exerting an inhibitory effect on the Harris pathway [39]. Consequently, this study found no significant differences in higher alcohol content at varying sugar concentrations during the early stage of fermentation. Aminotransferases can be categorized into two major types based on the amino acids they catalyze: one type comprises branched-chain amino acid transaminases encoded by the *BAT1* and *BAT2* genes [40], while the other type includes aromatic amino acid transaminases encoded by the *ARO8* and *ARO9* genes [41]. In this study, the transcription levels of the *BAT1*, *BAT2*, *ARO8*, and *ARO9* genes exhibited an upregulation trend during the early fermentation stage across various sugar concentrations. When examining the impact of the *BAT1* and *BAT2* genes on the higher alcohol metabolism of *S. cerevisiae*, it was observed that the individual knockout of either the *BAT1* or *BAT2* gene significantly decreased the production of higher alcohols [42]. The overexpression of the *BAT1* and *BAT2* genes enhances the yeast’s ability to produce higher alcohols, with *BAT2* overexpression resulting in a more pronounced increase in higher alcohol yield [43]. The *ARO8* gene primarily facilitates the deamination reaction of phenylalanine, and its overexpression promotes the synthesis of 2-phenylethanol [44]. Research has shown that the overexpression of either the *ARO8* or *ARO9* gene can further enhance the yield of 2-phenylethanol.

In the late stages of fermentation, the scarcity of available free amino nitrogen in the fermentation broth hampers the supply of amino acids, thereby inhibiting the conversion of α-keto acids produced by yeast metabolism into their corresponding amino acids. The insufficient availability of amino acid leads to the accumulation of α-keto acids, which are subsequently converted into higher alcohols. During this phase, the Harris pathway is activated, resulting in the synthesis of a substantial amount of higher alcohols. The primary genes involved in pyruvate synthesis are the *LEU* genes, which play a crucial role in leucine biosynthesis. Research indicates that the knockout of the *LEU2* gene significantly increases isobutanol production [45]. In the current study, the transcription levels of the *LEU* genes decrease as sugar concentration increases during the late stages of fermentation, culminating in a rapid rise in higher alcohol content.

According to the theory of metabolic regulation, enhancing the activity of decarboxylases and alcohol dehydrogenases while inhibiting aldehyde dehydrogenases can significantly increase the production of higher alcohols; conversely, decreasing the former and increasing the latter can lead to the opposite effect. Decarboxylases encoded by *PDC* genes catalyze the decarboxylation of α-keto acids, leading to the formation of aldehydes [46], Subsequently, these aldehydes are reduced to their corresponding alcohols through the action of alcohol dehydrogenases encoded by *ADH* genes [47,48]. Additionally, aldehydes can be oxidized to their respective alcohols under the catalysis of aldehyde dehydrogenases encoded by *ALD* genes [49]. In the present study, the transcription levels of the *PDC1*, *ADH1*, and *ADH2* genes were upregulated during the late stage of fermentation as sugar concentration increased. In contrast, the transcription levels of the *ALD2/3/4/5* genes decreased during this phase of fermentation with rising sugar concentration, a trend that may contribute to the elevated content of higher alcohols under high-sugar conditions.

### 4.3. GRE3 Is Involved in the Production of Higher Alcohols

The Venn diagram revealed significant changes in *GRE3* expression at different sugar concentrations and fermentation stages. The expression of *GRE3* increased with an increase in sugar concentration and the fermentation process (Figure 4C). Research has demonstrated that *GRE3*, which relies exclusively on NADPH, functions as an aldose reductase capable of converting various aldoses into their corresponding sugar alcohols, owing to its broad substrate specificity. This enzyme can reduce glucose to sorbitol, a polyol that acts as an osmotic protectant [50,51]. Additionally, *GRE3* is involved in the metabolism of d-xylose, arabinose, and galactose, catalyzing their conversion into the respective polyols [52]. Furthermore, high expression levels of *GRE3* have been associated with increased erythritol content and improved sugar alcohol conversion rates, likely contributing to the maintenance of intracellular redox balance [53,54]. Therefore, we hypothesize that the depletion of nitrogen sources inhibits the metabolic conversion pathway from pyruvate to α-ketoglutarate within amino acid metabolism. This alteration redirects the carbon flux of glycolysis toward the pentose phosphate pathway. Specifically, under high-sugar conditions, *GRE3* expression is significantly upregulated, compensating for redox imbalance through the synthesizing of polyols. Consequently, yeast cells may no longer utilize amino acids for energy and must rely on alternative pathways, such as the pentose phosphate pathway, to meet their energy demands. The production of polyols aids in balancing NADPH consumption during synthesis, enabling yeast cells to maintain redox homeostasis and survive. This phenomenon may elucidate why *GRE3* overexpression is associated with increased levels of higher alcohols.

## 5. Conclusions

In this study, it was demonstrated that high-sugar fermentation increases the concentration of higher alcohols through transcriptome sequencing and gene enrichment analysis. First, *S. cerevisiae* enhances the utilization of carbon sources, leading to the production of higher alcohols. Second, high sugar concentrations may promote the expression of *GRE3*, resulting in increased consumption of NADPH by yeast to maintain redox balance and facilitate the production of higher alcohols. *GRE3* was the only gene screened for significant changes at different sugar concentrations during each fermentation stage. The verification results showed that this gene has a significant effect on the production of higher alcohols and has a regulatory effect on higher alcohol metabolism. This study demonstrates that *S. cerevisiae* regulates NADPH metabolic flow via the *GRE3* gene during high-sugar fermentation, facilitating the synthesis of higher alcohols. This finding elucidates the coupling mechanism between enhanced carbon source utilization and redox balance. Building on this discovery, engineered strains tolerant to high sugar concentrations (>300 g/L) can be developed through synthetic biology strategies. This approach involves co-regulating *GRE3* and redox-related genes, constructing multi-gene co-expression systems, and precisely editing the metabolic network using gene-editing technologies to achieve targeted regulation of higher alcohol production. However, this study is limited to standard laboratory strains and has not verified the conservation of *GRE3* function in industrial strains.

## Figures and Tables

**Table 1:** Physicochemical indexes of *S. cerevisiae* LFE11225 at different fermentation stages with three sugar concentrations.

Metabolite	Early			Middle			Late			End		
	200 g/L	240 g/L	280 g/L	200 g/L	240 g/L	280 g/L	200 g/L	240 g/L	280 g/L	200 g/L	240 g/L	280 g/L
Residual sugar (g/L)	151.50 ± 3.02 ^a^	188.11 ± 2.33 ^b^	227.02 ± 7.45 ^c^	104.11 ± 5.29 ^a^	124.11 ± 6.35 ^b^	164.71 ± 6.10 ^c^	11.89 ± 1.99 ^a^	22.88 ± 3.04 ^b^	39.88 ± 6.98 ^c^	1.73 ± 0.49 ^a^	2.58 ± 0.33 ^b^	1.73 ± 1.06 ^a^
Ethanol (% *v*/*v*)	2.63 ± 0.03 ^a^	2.77 ± 0.12 ^a^	2.63 ± 0.30 ^a^	5.07 ± 0.23 ^a^	6.20 ± 0.36 ^b^	4.83 ± 2.47 ^ab^	10.47 ± 0.12 ^a^	12.67 ± 0.40 ^b^	11.80 ± 2.00 ^ab^	11.23 ± 0.29 ^a^	13.97 ± 0.29 ^b^	15.10 ± 0.14 ^c^
Higher alcohol (mg/L)	130.01 ± 7.56 ^c^	144.70 ± 3.26 ^b^	155.55 ± 1.97 ^a^	227.22 ± 4.70 ^c^	273.89 ± 17.62 ^b^	340.80 ± 15.92 ^a^	428.75 ± 6.63 ^c^	569.51 ± 6.92 ^b^	599.50 ± 2.68 ^a^	486.60 ± 8.90 ^c^	571.62 ± 14.31 ^b^	601.92 ± 9.31 ^a^
Total acid (g/L)	9.94 ± 0.9 ^a^	10.41 ± 0.93 ^ab^	11.78 ± 0.59 ^b^	8.06 ± 0.55 ^a^	8.40 ± 0.82 ^ab^	9.54 ± 0.38 ^b^	6.89 ± 0.42 ^a^	7.91 ± 0.17 ^a^	7.50 ± 0.89 ^a^	6.03 ± 0.12 ^a^	6.95 ± 0.05 ^b^	6.90 ± 0.09 ^b^
pH	3.22 ± 0.04 ^a^	3.25 ± 0.01 ^a^	3.25 ± 0.01 ^a^	3.13 ± 0.01 ^a^	3.20 ± 0.03 ^b^	3.15 ± 0.01 ^a^	3.07 ± 0.06 ^a^	3.14 ± 0.01 ^a^	3.14 ± 0.03 ^a^	3.17 ± 0.02 ^a^	3.30 ± 0.02 ^c^	3.24 ± 0.02 ^b^
Glycerol (g/L)	1.73 ± 0.12 ^a^	2.07 ± 0.12 ^b^	1.73 ± 0.12 ^a^	2.73 ± 0.42 ^a^	3.33 ± 0.31 ^b^	4.47 ± 0.23 ^c^	4.00 ± 0.20 ^a^	4.93 ± 0.12 ^b^	5.93 ± 0.76 ^c^	4.33 ± 0.31 ^a^	5.33 ± 0.12 ^b^	6.20 ± 0.72 ^c^
Citrate (g/L)	0.7 ± 0.06 ^a^	0.66 ± 0.05 ^a^	0.70 ± 0.06 ^a^	0.70 ± 0.04 ^a^	0.68 ± 0.04 ^a^	0.68 ± 0.04 ^a^	0.43 ± 0.02 ^a^	0.45 ± 0.02 ^a^	0.43 ± 0.05 ^a^	0.45 ± 0.06 ^a^	0.40 ± 0.04 ^a^	0.39 ± 0.00 ^a^
Succinate (g/L)	0.00 ± 0.00 ^a^	0.00 ± 0.00 ^a^	0.00 ± 0.00 ^a^	0.00 ± 0.00 ^a^	0.00 ± 0.00 ^a^	0.00 ± 0.00 ^a^	0.75 ± 0.02 ^a^	0.82 ± 0.20 ^a^	0.69 ± 0.24 ^a^	0.61 ± 0.04 ^a^	0.69 ± 0.02 ^a^	0.63 ± 0.03 ^a^
Lactate (g/L)	0.00 ± 0.00 ^a^	0.00 ± 0.00 ^a^	0.00 ± 0.00 ^a^	0.00 ± 0.00 ^a^	0.00 ± 0.00 ^a^	0.00 ± 0.00 ^a^	0.17 ± 0.02 ^a^	0.18 ± 0.03 ^a^	0.18 ± 0.02 ^a^	0.20 ± 0.02 ^a^	0.17 ± 0.01	0.20 ± 0.02 ^a^
Malate (g/L)	7.02 ± 0.57 ^a^	7.64 ± 0.67 ^ab^	8.18 ± 0.05 ^b^	5.61 ± 0.26 ^a^	6.31 ± 0.88 ^ab^	6.88 ± 0.35 ^b^	3.00 ± 0.43 ^a^	3.79 ± 0.05 ^b^	4.07 ± 0.30 ^b^	2.39 ± 0.05 ^a^	2.51 ± 0.03 ^a^	2.53 ± 0.11 ^a^
Pyruvate (g/L)	0.07 ± 0.02 ^a^	0.09 ± 0.01 ^a^	0.08 ± 0.03 ^a^	0.11 ± 0.02 ^a^	0.10 ± 0.02 ^a^	0.12 ± 0.01 ^a^	0.10 ± 0.01 ^a^	0.12 ± 0.00 ^a^	0.10 ± 0.02 ^a^	0.07 ± 0.01 ^a^	0.06 ± 0.00 ^a^	0.05 ± 0.01 ^b^
Acetate (g/L)	0.00 ± 0.00 ^a^	0.00 ± 0.00 ^a^	0.00 ± 0.00 ^a^	0.00 ± 0.00 ^a^	0.00 ± 0.00 ^a^	0.00 ± 0.00 ^a^	0.36 ± 0.16 ^a^	0.70 ± 0.15 ^a^	0.52 ± 0.17 ^a^	0.50 ± 0.08 ^a^	0.72 ± 0.09 ^b^	0.86 ± 0.40 ^b^

Note: Different letters in the same line indicate significant differences among samples *p* < 0.05.

## Data Availability

The original contributions presented in this study are included in the article/Supplementary Materials. Further inquiries can be directed to the corresponding author.

## Supplementary Materials

The following supporting information can be downloaded at: https://www.mdpi.com/article/10.3390/foods14091476/s1, Figure S1: The result of fermentation rate and important metabolite contents at three initial sugar concentrations throughout the fermentation process. (A): The fermentation rate of LFE1225. (B): LFE1225 CO_2_ weight loss. (C): The organic acid, glycerol, and alcohol contents of LFE1225. Table S1: Strains and plasmids used in this experiment. Table S2: Quality assessment of sequencing data. Table S3: Number of differentially expressed genes compared with different sugar concentrations.
