# Supplementary material for: Transcriptomic Identification of Core Regulatory Genes for Higher Alcohol Production in Saccharomyces cerevisiae at Different Sugar Concentrations in Wine Fermentation
Source: Foods. 2025 Apr 23;14(9):1476. doi: 10.3390/foods14091476 (PMC12071402; doi:10.3390/foods14091476)
Supplement: Supplementary file 1 [file foods-14-01476-s001.zip › foods-3540635-supplementary.pdf]

## Supplementary Materials:

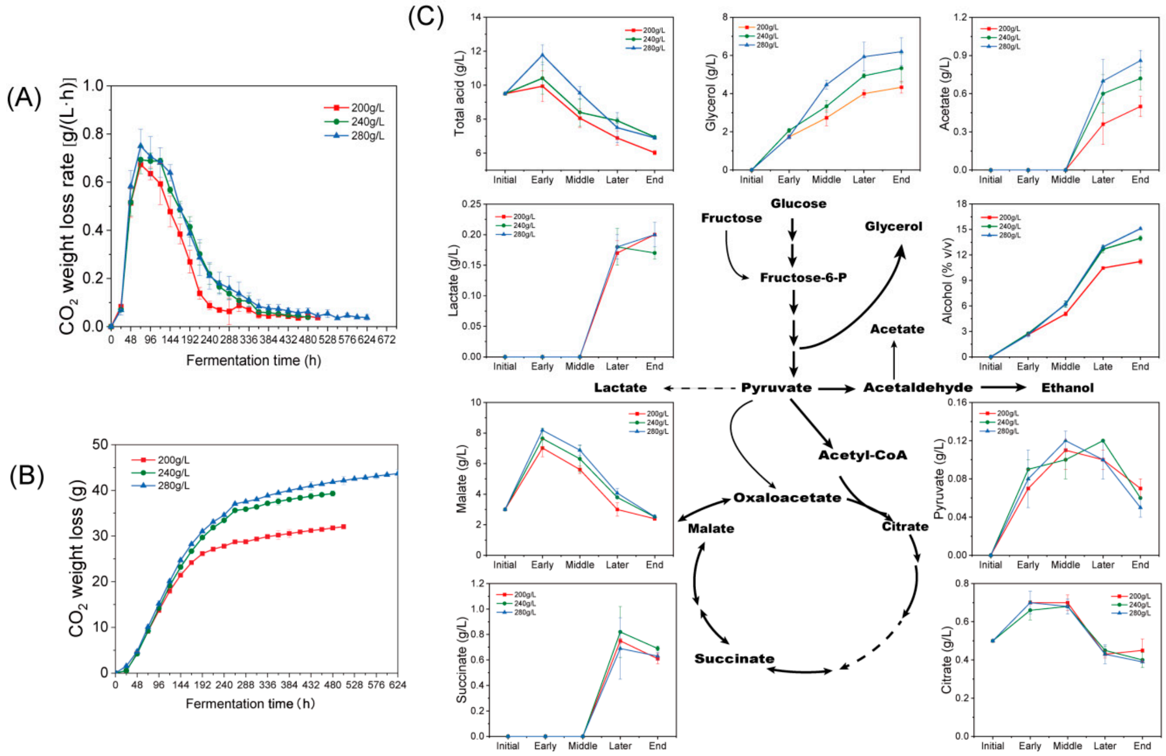


**Figure S1** The result of fermentation rate and important metabolite contents at three initial sugar concentrations throughout the fermentation process. (A): The fermentation rate of LFE1225. (B): LFE1225 $CO_2$ weight loss. (C): The organic acid, glycerol, and alcohol contents of LFE1225.

**Table S1** Strains and plasmids used in this experiment

| | Description | Source |
|---|---|---|
| *Strains* | | |
| LFE1225 | Wild-type diploid *S. cerevisiae* strain | Laboratory storage |
| LFE1225ΔURA3 | LFE1225 *URA3* 121_131 insTAATAA | Laboratory storage |
| LFE1225ΔGRE3 | LFE1225 *GRE3* 42_49 insTAATAA | This study |
| LFE1225 pY26 | LFE1225 with pY26 plasmid | This study |
| LFE1225 GRE3 | LFE1225 with pY26-*GRE3* plasmid | This study |
| *Plasmids* | | |
| pY26 | pY26TEF-GPD *S. cerevisiae* high copy bidirectional expression plasmid | Laboratory storage |
| pRCC-K | Expression of Cas9 and gRNA cassette in *S. cerevisiae*; Resistance. | Laboratory storage |
| pY26-GRE3 | *GRE3* overexpression vector | This study |
| pRCC-K-GRE3 | *GRE3* knockout vector | This study |
| *Primer* | | |
| sgF1 | GAAAGATAAATGATCGGATCggtctgaaaatgcccctagtGTTTTAGAGCTAGAAATAGC | |
| sgF2 | CCTCGAGTCATGTAATTAGT | |
| G-F | ACTAATTACATGACTCGAGGttaccgctcgaagaaagtgg | |
| G-Rm | ttccagcaccctaagcttattagggcattttcagaccgtt | |
| G-Fm | aacggtctgaaaatgccctaataagcttagggtgctggaa | |
| G-R | GAAGAGAAAGGTTTAAGTCGAgtggcaccgcaatcattact | |
| G-A | ggattctagaactagtggatcccagtgcaatccttcaagacg | |
| G-D | cgataagcttgatatcgaattatacagcatcggaatgaggg | |

***Quality assessment of sequencing data***

Samples from Early, middle and late stages were collected at 200, 240 and 280 g/L sugar concentrations. The nine independent cDNA libraries (200Early-FA, 200Middle-FB, 200Late-FC, 240Early-FA, 240Middle-FB, 240Late-FC, 280Early-FA, 280Middle-FB and 280Late-FC) constructed for high-throughput sequencing have produced 2.34~3.60 billion bp raw data, respectively. After rigorous quality checks and data filtering, the filtered sequenced clean data were compared with the *S. cerevisiae* S288C genome. The results of the comparison of the reads with reference genes are shown in Table S2. The percentage of sequences mapped to the genome under nine conditions ranged from 90.40 % to 96.52 %. These data suggest that the throughput and sequencing quality is sufficiently high to allow further analysis.

Table S2 Quality assessment of sequencing data

| Sample | RawData(bp) | Total_Mapped(%) |
|---|---|---|
| T200-A-1 | 2794385700 | 17141511 (95.63%) |
| T200-A-2 | 3265883700 | 19270355 (95.54%) |
| T200-A-3 | 2934425400 | 18103942 (95.47%) |
| T240-A-1 | 3551109000 | 22261525 (96.15%) |
| T240-A-2 | 3287197200 | 20026591 (96.22%) |
| T240-A-3 | 2800554600 | 17414174 (96.21%) |
| T280-A-1 | 2343638400 | 14443695 (95.80%) |
| T280-A-2 | 3354713700 | 21033707 (95.43%) |
| T280-A-3 | 3312648900 | 20660663 (94.91%) |
| T200-B-1 | 3138864000 | 18140924 (96.52%) |
| T200-B-2 | 2932266600 | 16790837 (96.35%) |
| T200-B-3 | 3072270000 | 18314931 (96.29%) |
| T240-B-1 | 2817833700 | 15657762 (95.73%) |
| T240-B-2 | 2902911600 | 16001183 (95.65%) |
| T240-B-3 | 2723486700 | 14826036 (96.04%) |
| T280-B-1 | 3552742800 | 6476240 (90.97%) |
| T280-B-2 | 3556862700 | 18886544 (94.36%) |
| T280-B-3 | 3334237500 | 16968175 (94.45%) |
| T200-C-1 | 2778234900 | 13441302 (95.29%) |
| T200-C-2 | 3602215200 | 18151185 (94.91%) |
| T200-C-3 | 3310698600 | 15655180 (94.92%) |
| T240-C-1 | 3101318400 | 15987716 (95.09%) |
| T240-C-2 | 3085438500 | 15193052 (95.06%) |
| T240-C-3 | 3011685300 | 10089233 (94.73%) |
| T280-C-1 | 3492014700 | 12053805 (93.23%) |

| Sample | RawData(bp) | Total_Mapped(%) |
|---|---|---|
| T280-C-2 | 3113328000 | 7973286 (90.40%) |
| T280-C-3 | 3586360200 | 11340635 (92.34%) |

Table S3 Number of differentially expressed genes compared with different sugar concentrations

| Pairs | Up | Down | Total |
|---|---|---|---|
| FA200vs240 | 13 | 91 | 104 |
| FA200vs280 | 290 | 302 | 529 |
| FA240vs280 | 35 | 28 | 63 |
| FB200vs240 | 85 | 66 | 151 |
| FB200vs280 | 239 | 332 | 571 |
| FB240vs280 | 24 | 23 | 47 |
| FC200vs240 | 110 | 373 | 483 |
| FC200vs280 | 156 | 244 | 400 |
| FC240vs280 | 87 | 122 | 209 |
